# Endogenous IL-22 Plays a Dual Role in Arthritis: Regulation of Established Arthritis via IFN-γ Responses

**DOI:** 10.1371/journal.pone.0093279

**Published:** 2014-03-27

**Authors:** Shivali Justa, Xiaoqun Zhou, Sujata Sarkar

**Affiliations:** Section of Rheumatology, Department of Medicine, and the Arizona Arthritis Center, University of Arizona, Tucson, Arizona, United States of America; University Hospital Jena, Germany

## Abstract

**Objective:**

IL-22 is elevated in patients with inflammatory arthritis and correlates with disease activity. IL-22 deficient mice have reduced incidence of arthritis. Recombinant IL-22 restrains progression of arthritis via increase in IL-10 responses when administered prior to onset of arthritis. These findings imply a possible dual role of IL-22 in inflammatory arthritis depending on the phase of arthritis. Experiments outlined here were designed to elucidate the contribution of endogenous IL-22 before and after the onset of arthritis.

**Methods:**

Collagen induced arthritis (CIA) was induced in DBA1 or IFN-γ deficient mice following immunization with collagen and complete Freund's adjuvant. Anti-IL-22 antibody or isotype control were administered prior to or after onset of arthritis and disease progression assessed by clinical scoring and histopathology. IL-22, IL-17 and IFN-γ responses were measured by ELISA and flowcytometry. Anti-collagen antibody responses were analyzed by ELISA. Expression of IL-22R1 in CD4+ cells was elucidated by flowcytometry and real time PCR.

**Results:**

Collagen specific IL-22 responses were expanded during arthritis and IL-22 producing cells were discrete from IL-17 or IFN-γ producing cells. Neutralization of IL-22 after onset of arthritis resulted in significant increase in Th1 responses and significantly reduced severity of arthritis. CD4+ cells from arthritic mice showed increased surface expression of IL-22R1. In vitro, CD4+T cells cultured with antigen presenting cells in the presence or absence of IL-22 suppressed or induced IFN-γ, respectively. The protective effect of anti-IL-22 was reversed in IFN-γ deficient mice. Moreover, administration of anti-IL-22 prior to onset of arthritis augmented arthritis severity.

**Conclusion:**

We show for the first time that IL-22 plays a dual role: protective prior to the onset of arthritis and pathogenic after onset of arthritis. The pathogenic effect of IL-22 is dependent on suppression of IFN-γ responses. IL-17 responses remained unchanged with the administration of anti-IL22 antibody. IL-22R1 is upregulated on CD4+T cells during arthritis and regulates IFN-γ in T cells.

## Introduction

IL-22, belongs to the IL-10 family of cytokines. IL-22 is primarily produced by CD4 T cells, NK cells, and LTi cells [1]. The receptor for IL-22 is a heterodimeric receptor composed of the IL-22R1 subunit exclusive to IL-22 and the IL-10R2 subunit which is the shared subunit with other members of the IL-10 family of cytokines [2]. IL-22 is pathogenic in psoriasis and protective in inflammatory bowel disease, hepatitis, Klebsiella pneumonia, myocarditis, ulcerative colitis, airway inflammation and autoimmune allergic asthma [3], [4], [5], [6], [7], [8], [9], [10], [11], [12].

In rheumatoid arthritis (RA), IL-22 responses are increased in peripheral blood and joints, IL-22 induces RANKL, and the magnitude of IL-22 response correlates with inflammatory markers (ESR and CRP), RA disease activity scores and degree of bone damage [13], [14], [15], [16], [17], [18]. IL-22 knock-out mice have reduced incidence of collagen induced arthritis (CIA; the most widely used model of autoimmune inflammatory arthritis) [19]. In our previous study we reported that administration of recombinant IL-22 prior to the onset of arthritis reduces the severity of subsequent arthritis via increase in IL-10, implying a possible protective role of IL-22 during this phase [20]. Put together, these findings imply that IL-22 may play a dual role in arthritis depending on the phase of arthritis. In this study we have administered neutralizing anti-IL-22 antibody prior to and after onset of arthritis to investigate the possible dual role and evaluate the mechanism underlying the pathogenic function of IL-22 during arthritis.

Our studies show that neutralization of endogenous IL-22 after onset of arthritis is associated with reduction in the severity of arthritis supportive of a pathogenic role of IL-22 in the presence of inflammation. IL-22R1 is upregulated in T cells during arthritis and IL-22 regulated IFN-γ in-vitro and in-vivo. The pathogenic effect of IL-22 is abolished in IFN-γ deficient mice. Additionally, neutralization of IL-22 prior to onset of arthritis resulted in increased incidence and severity of arthritis, supportive of a protective effect of IL-22 prior to onset of arthritis.

## Materials and Methods

### Mice

Male DBA/1 mice and female B6.129S7-ifng^tm1Ts^ (B6.IFNγKO), 8–10 weeks of age, were obtained from Taconic farms (Hudson, NY) and Jackson Laboratory respectively and were housed in specific pathogen free condition at the University of Arizona animal care facility. All procedures were approved by the University Committee for the Use and Care of Animals of the University of Arizona. Euthanasia was performed using isoflurane inhalation and all efforts were made to minimize suffering.

### Ethics statement

Experiments involving animals were carried out in accordance with institutional guidelines under protocol (08-063) approved by the Animal Care and Use Committee of the University of Arizona.

### Collagen induced arthritis

Mice were immunized with chicken type II collagen emulsified with Complete Freund adjuvant (CFA). Preparation, immunization and clinical scoring were done as previously reported [21].

### IL-22 neutralization before onset of arthritis

Male DBA/1 mice were immunized with collagen and CFA and divided into two groups. One group received neutralizing antibody to IL-22 and another group received mouse IgG isotype control antibody. Mice were scored for arthritis every other day. Anti-IL-22 antibody (clone 8E11, a kind gift from Dr. Wenjun Ouyang, Genentech, San Francisco, CA) or mouse IgG isotype control antibody (Biolegend) were administered intraperitoneally at 100 ug/day/mouse for 8–10 days, starting day 18 post immunization.

### IL-22 neutralization after onset of arthritis

Male DBA/1 or female B6.129S7-ifng^tm1Ts^ (B6.IFNγKO) were scored for arthritis every other day, starting around day 21 following immunization with type II collagen and CFA. Mice with clinical scores of ≥1 and with arthritis duration of 4 days were randomized to receive either neutralizing antibody to IL-22 or mouse IgG isotype control antibody intraperitoneally at 100 ug/day/mouse for 8–12 days.

### Tissue harvest and assays

Mice were euthanized with overdosage of inhalational anesthesia. Blood was collected by cardiac puncture, serum separated and stored at −80°C for the analysis of anti-collagen antibodies at a later date. Single cell suspension of spleen and draining inguinal lymph nodes were stimulated at (5×10^6^/ml) with 5 ug/ml of anti-CD3 (clone 1452C11) (Biolegend, USA) for 3 days or 100 ug/ml of chicken collagen for 7 days. Culture supernatants were collected for cytokine analysis at a later date. Paws were dissected at the fur line, cut into small pieces after removal of overlying skin and then digested with collagenase to make a single cell suspension as previously reported [20]. Paw single cell suspension (5×10^6^/ml) was restimulated in a manner similar to that described above for cells from lymphoid organs, and culture supernatants were frozen for cytokine analysis at a later date.

Splenocytes from arthritic mice were enriched for CD4+, CD19+ or CD11c+ cells by positive selection using anti-CD4 or anti-CD19 or anti-CD11c microbeads (Miltenyi Biotech, USA). The purity of the enriched CD4+, CD19+, and CD11c+ cells were 96%, 93% and 95%, respectively. CD4+ cells were then co-cultured with CD19+ cells at ratio 1∶1 (5×10^6^/ml) or with CD11c+ cells at ratio 5∶1 (5×10^6^/ml) and stimulated with collagen, in the presence or absence of recombinant IL-22 (100 ng/ml, Insight Genomics, USA), or anti-IL-22 antibody (10 ug/ml) or mouse IgG isotype control (10 ug/ml, Biolegend) for 7 days. Culture supernatants were collected for cytokine analysis at a later date.

### Cytokine measurements by ELISA

IL-22, IFN-γ, IL-17A and IL-10 were determined using commercially available ELISA kits according to manufacturer's protocols (R&D Systems for the IL-22 kit; BioLegend for others).

### Flow-cytometry

For surface staining anti-CD4 (clone GK1.5, eBioscience), anti-IL22R1 (clone 496514, R&D sytems) or relevant isotype control antibodies were used. For intracellular flowcytometry, splenocytes or paws cells were stimulated with PMA(5 ng/ml) and Ionomycin(500 ng/ml) (Sigma-Aldrich, USA) and Brefeldin A (Biolegend) for 6 hours, prior to staining with anti-IL-17A (clone TC11-18H10.1, Biolegend), anti-IFN-γ (clone XMG1.2, eBioscience), or anti-IL-22 (clone IL-22JOP, eBioscience) antibodies or relevant isotype controls. Data was acquired with BD LSR and analyzed using Flow-Jo software.

### Gene expression analysis of IL-22R1, IL-10R2 and IL-10 by real time PCR

Enriched CD4+, CD19+ and CD11c+ cells from naïve mice, from mice 2 weeks following immunization with collagen (initiation phase) and arthritic mice were used for preparation of RNA using RNA columns (Qiagen, USA). RNA was then transcribed to cDNA using a reverse transcription kit (Applied Biosystems). The expression patterns of IL-22R1 and IL-10R2 were analyzed by Taqman PCR using primers and probes from Applied Biosystems. For some experiments IL-10 expression was measured by realtime PCR in splenocytes using primers and probes from Applied Biosystems. GAPDH was used as internal controls. Data was analyzed using SDS software.

### Measurement of Abs against type II collagen

Anti-collagen antibodies were measured in mouse serum as outlined previously [20].

### Tissue histology and scoring

Paws were collected at time of harvest and processed for histopathologic scoring as outlined previously [20].

### Statistical analysis

Statistical analysis was performed using GraphPad Prism. Data are presented as mean +/− SEM. Significance was analyzed using the Student's t-test or Mann-Whitney test. P value less than 0.05 was considered significant.

## Results

### Induction of collagen specific IL-22 response during arthritis

**Figure 1:**
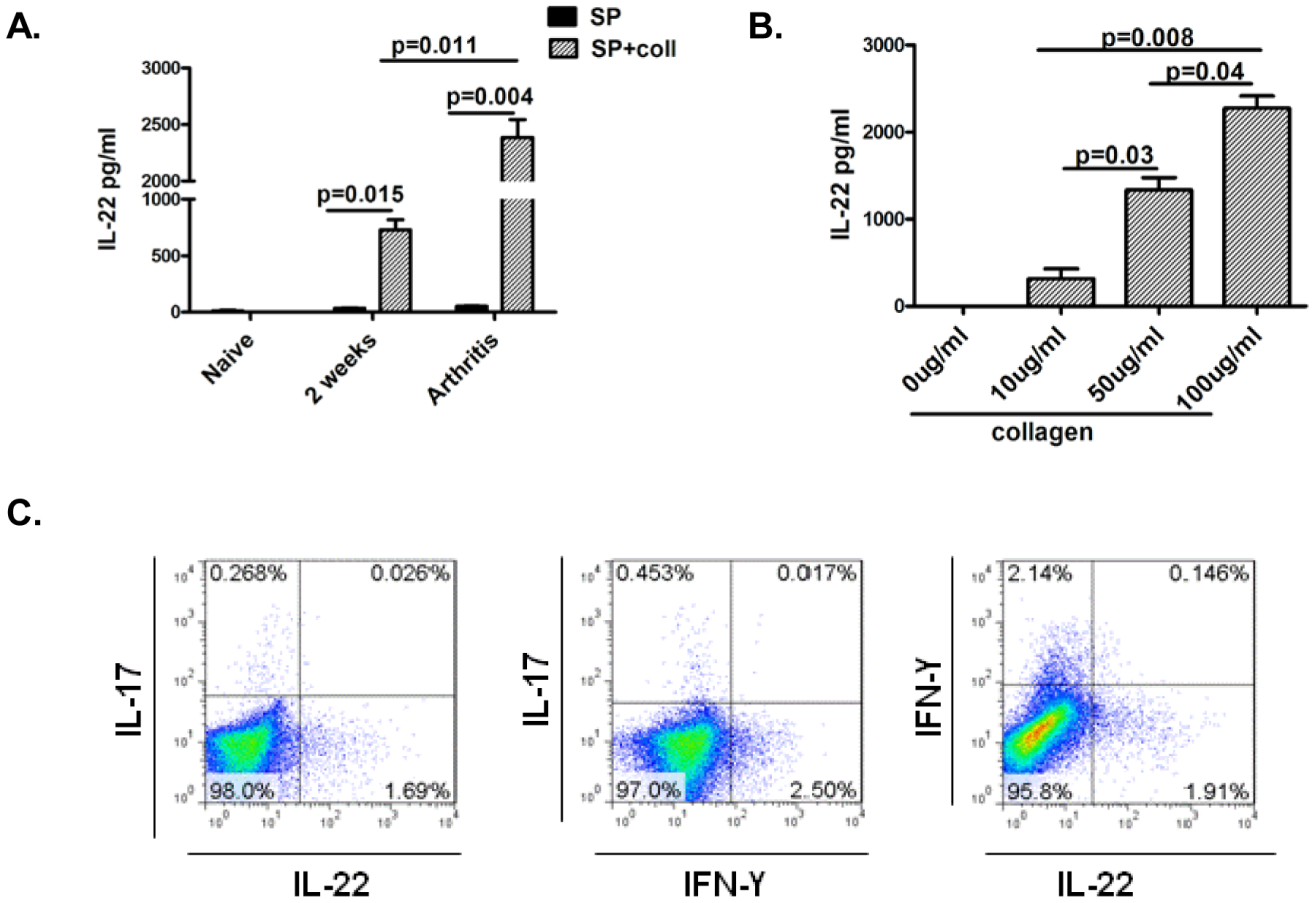
Induction of IL-22 response during arthritis. **A:** DBA mice were immunized with collagen and CFA. Splenocytes (5×10^6^/ml) from naïve mice, mice during initiation phase (2 weeks following immunization with collagen and CFA) or arthritic mice were stimulated with collagen (100 ug/ml) for 7 days or unstimulated. Supernatants were analyzed for IL-22 by ELISA. Data shown is representative of 3 independent experiments with 3 mice per experiment. **B:** Splenocytes (5×10^6^/ml) from arthritic mice were stimulated with varying concentrations of collagen (0, 10, 50 or 100 ug/ml) for 7 days and IL-22 was measured in supernatant by ELISA. Data shown is representative of 3 independent experiments with 3 mice per experiment. **C:** Splenocytes from arthritic mice were stimulated with PMA/ionomycin and Brefeldin A for 6 hours, stained intra-cellularly for IL-17, IFN-γ or IL-22 and analyzed by flow-cytometry. Data shown is gated on mononuclear cells based on forward and side scatter. Data is representative of 3 independent experiments with 3 mice per experiment.

A variety of immune cells including T cells, NK cells or LTi cells have been reported to produce IL-22 in response to innate and/or antigen specific stimuli [1]. Previous studies have reported that IL-22 is induced in lymphoid organs during arthritis and produced by CD4 T cells and/or CD49b+ cells [20]. In order to elucidate collagen specific IL-22 response during arthritis, splenocytes from naïve mice, mice from the initiation phase or arthritic mice were stimulated with collagen and IL-22 response was measured in culture supernatants by ELISA. Collagen restricted IL-22 response was augmented during arthritis (Fig. 1A). Furthermore, IL-22 levels in collagen restimulation cultures increased in a dose dependent fashion (Fig. 1B). In order to elucidate if IL-22 producing cells co-produced IL-17 or IFN-γ, splenocytes from arthritic mice were analyzed for IL-22, IL-17 or IFN-γ by intracellular flowcytometry. Data in figure 1C shows that IL-22 producing cells were distinct from IL-17 or IFN-γ producing cells. Similar results were obtained with draining inguinal lymph nodes from arthritic mice (data not shown).

Joint specific IL-22 responses were elucidated by single cell culture and flow cytometry of joint cells. Single cell suspension of joint cells was stimulated with collagen for 7 days. IL-22, IL-17 or IFN-γ could not be detected in the collagen stimulated cultures of arthritic joints by ELISA. Neither, IL-17, IFN-γ nor IL-22 was detectable when collagen was used for stimulation of single cell suspension of arthritic joints, probably due to the low numbers of antigen specific cells in arthritic joints. To analyze if polyclonal stimulation would elicit IL-22 responses in the joints, single cell suspension of arthritic joints were stimulated with anti-CD3 for 3 days and supernatants analyzed for IL-22, IL-17 or IFN-γ by ELISA. IL-17 was detectable whereas IL-22 and IFN-γ remained undetectable (Fig. S1A). Single cell suspensions of arthritic joints were then analyzed for production of IL-22, IL-17 and IFN-γ by flow-cytometry. Data in figure S1B shows that although IL-17 producing cells were detectable in the arthritic joints, there was a paucity of IFN-γ or IL-22 producing cells in the joints. Moreover, IL-17 producing cells were present in the CD4+ subset (Fig. S1C).

Put together, these findings suggest that during arthritis IL-22 producing cells and the IL-22 response is more abundant in lymphoid organs in comparison to arthritic joints.

### Neutralization of IL-22 after onset of arthritis reduces severity of arthritis

**Figure 2:**
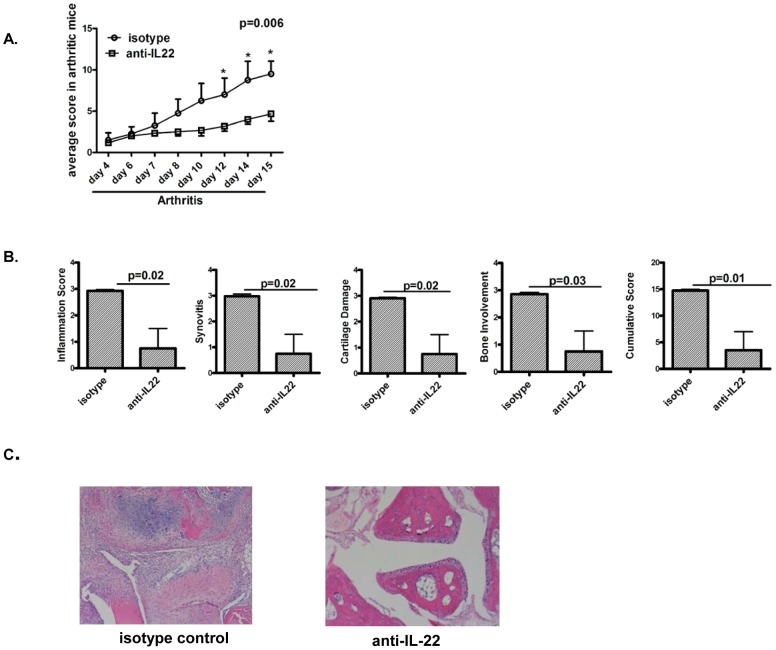
Neutralization of IL-22 after onset of arthritis reduces severity of arthritis. **A.** 16 DBA mice were immunized with collagen and CFA. Mice with total score ≥1 and arthritis duration of ≥4 days were randomized to receive anti-IL-22 antibody (7–8 mice) or isotype control (7–8 mice) intraperitoneally at 100 ug/mouse/day for 10–12 days. Mice were scored for clinical arthritis every other day by a person blinded to interventions. Data shows cumulative arthritis score in 2 groups over time. Data shown is representative of 2 independent experiments. **B.** Histologic scoring of paws for various aspects of joint damage and inflammation assessed after H&E staining. Data is from 4 mice per group. **C.** Data shows the histology of hematoxylin and eosin staining of representative paws from the two groups of mice, magnification of 10×.

In order to evaluate the effector function of endogenous IL-22 after onset of joint inflammation, anti-IL-22 antibody was administered in arthritic mice. Mice with arthritis scores of > or  = 1 and arthritis duration of 4 days were randomized to receive anti-IL-22 antibody or isotype control antibody for a total of 10–12 days. As shown in figure 2A mice receiving anti-IL-22 antibody had lower joint inflammation scores than mice receiving isotype control antibody. In keeping with the lower clinical scoring, histology of the joints showed significantly lower scores of inflammation, synovitis, cartilage destruction and bone involvement in mice receiving anti-IL-22 antibody (Fig. 2B and C).

### Administration of anti-IL-22 antibody is associated with altered B and T cell responses

**Figure 3:**
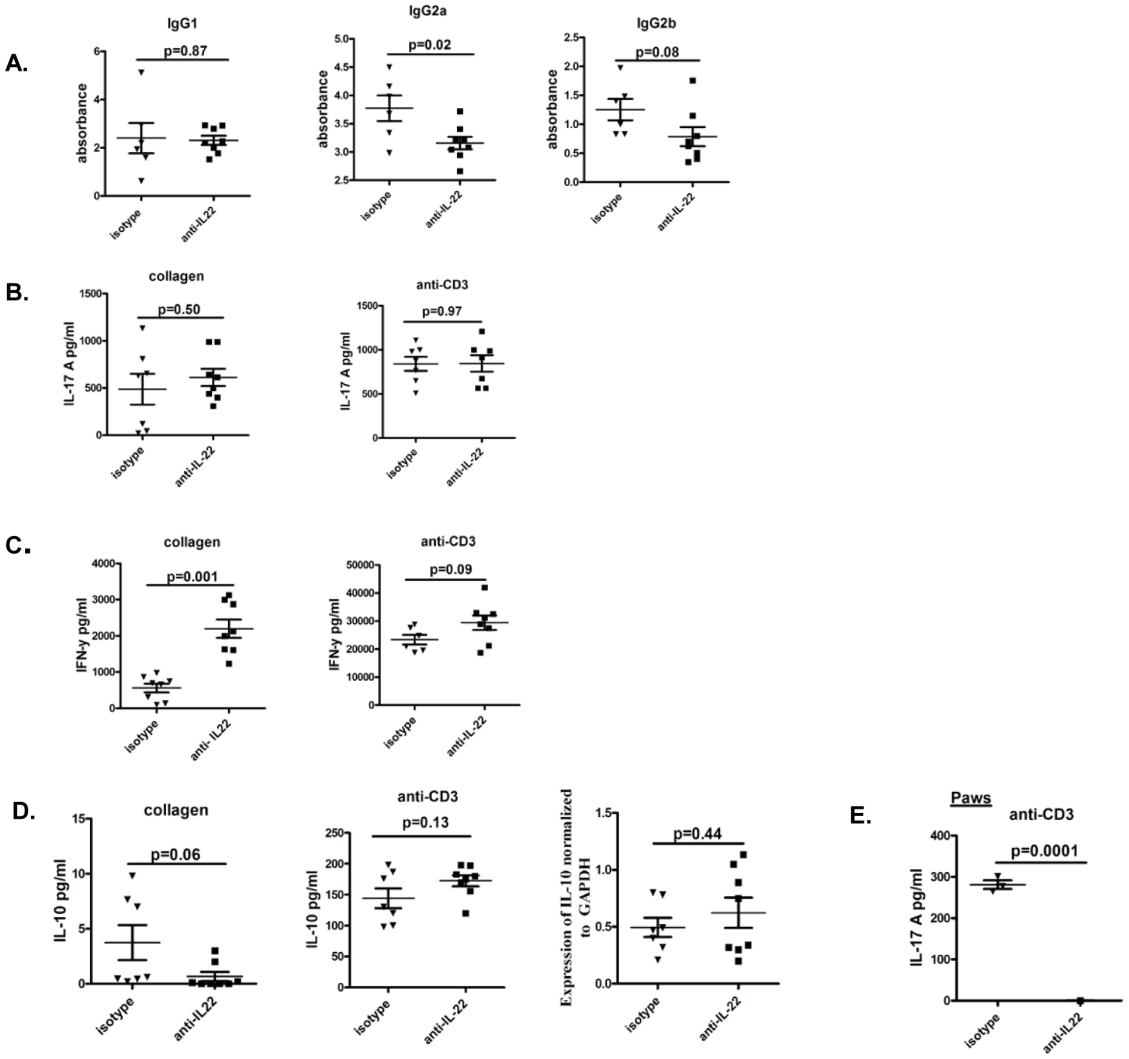
Administration of anti-IL-22 antibody is associated with altered B and T cell responses. Experimental design was the same as for figure 2. **A.** Sera from mice receiving either anti-IL-22 antibody or isotype control were analyzed for anti-collagen IgG1, IgG2a and IgG2b antibodies by ELISA. **B, C & D:** Splenocytes from mice receiving anti-IL-22 or isotype control were stimulated with anti-CD3 (5 ug/ml for 3 days) or collagen (100 ug/ml for 7 days) and IL-17A, IFN-γ and IL-10 were measured in culture supernatants by ELISA. For some experiments IL-10 was measured by real-time PCR from splenocytes of mice receiving anti-IL-22 antibody or isotype control and expressed as fold change over GAPDH. Data is representative of 2 independent experiments (8 mice per group) with each dot representing an individual mouse. **E:** Single cell suspensions of the paws from mice receiving anti-IL22 or isotype control were stimulated with anti-CD3 (5 ug/ml for 3 days) and IL-17A was measured in culture supernatants by ELISA. Each dot represents an individual mouse.

CIA is critically dependent on intact B and T cell responses. To investigate if the reduced severity of arthritis in anti-IL22 antibody treated mice was associated with altered anti-collagen antibody response levels of anti-collagen antibody were measured in sera of mice from both groups. Data in figure 3A shows that mice receiving anti-IL-22 antibody had somewhat reduced levels of anti-collagen IgG2a in comparison to mice receiving isotype control antibody. There were no significant differences in the levels of anti-collagen IgG1 and IgG2b between the two groups. In order to elucidate T cell responses, splenocytes from mice receiving anti-IL-22 or isotype control antibody were stimulated with anti-CD3 or collagen and IL-17A or IFN-γ analyzed in supernatants by ELISA. Figure 3B shows no significant differences in the levels of IL-17A responses in mice receiving anti-IL-22 or isotype control antibody. IFN-γ responses were significantly increased in mice receiving anti-IL-22 in comparison to mice receiving isotype control antibody (Fig. 3C). Such an increase was only observed with collagen specific stimulation and not seen when anti-CD3 was used for stimulation.

In our previous report administration of recombinant IL-22 prior to onset of joint inflammation increased IL-10 responses and restrained the progression of arthritis [20]. In this report neutralization of endogenous IL-22 after onset of arthritis lead to reduced joint inflammation (Fig. 2A). We wanted to evaluate if the increased IL-10 responses seen with the protective function of IL-22 was blunted or reversed during arthritis. To evaluate this we measured IL-10 expression by real-time PCR in splenocytes or stimulated splenocytes with anti-CD3 or collagen and measured IL-10 in culture supernatants, from the two groups of mice. There were no significant differences in the levels of IL-10 between the two groups (Fig. 3D).

In order to elucidate joint specific IL-17 and IFN-γ responses, single cell suspension of joints from mice receiving anti-IL-22 or isotype control were stimulated with anti-CD3 for 3 days or collagen for 7 days. IL-17 was only detectable in joint cells stimulated with anti-CD3 from mice from the isotype control group (Fig. 3E). No IL-17 was detectable from joint cells stimulated with anti-CD3 from anti-IL-22 group. This is in keeping with lower arthritis severity in mice receiving anti-IL-22 in comparison to isotype control.IL-17 was undetectable when joints cells were stimulated with collagen (data not shown), perhaps due to the low numbers of antigen specific cells in joints. IFN-γ remained undetectable whether joint cells were stimulated with anti-CD3 or collagen (data not shown).

Put together, antigen specific IL-22 response is induced with onset of arthritis. Neutralization of IL-22 after onset of joint inflammation is associated with less severe joint inflammation. This improvement in clinical response is associated with a significant increase in collagen specific IFN-γ responses, a modest reduction of anti-collagen IgG2a response, and no alteration of IL-17A or IL-10 response.

### Neutralization of IL-22 after onset of arthritis preferentially increases the frequency of Th1 cells in lymphoid organs but not in joints

**Figure 4:**
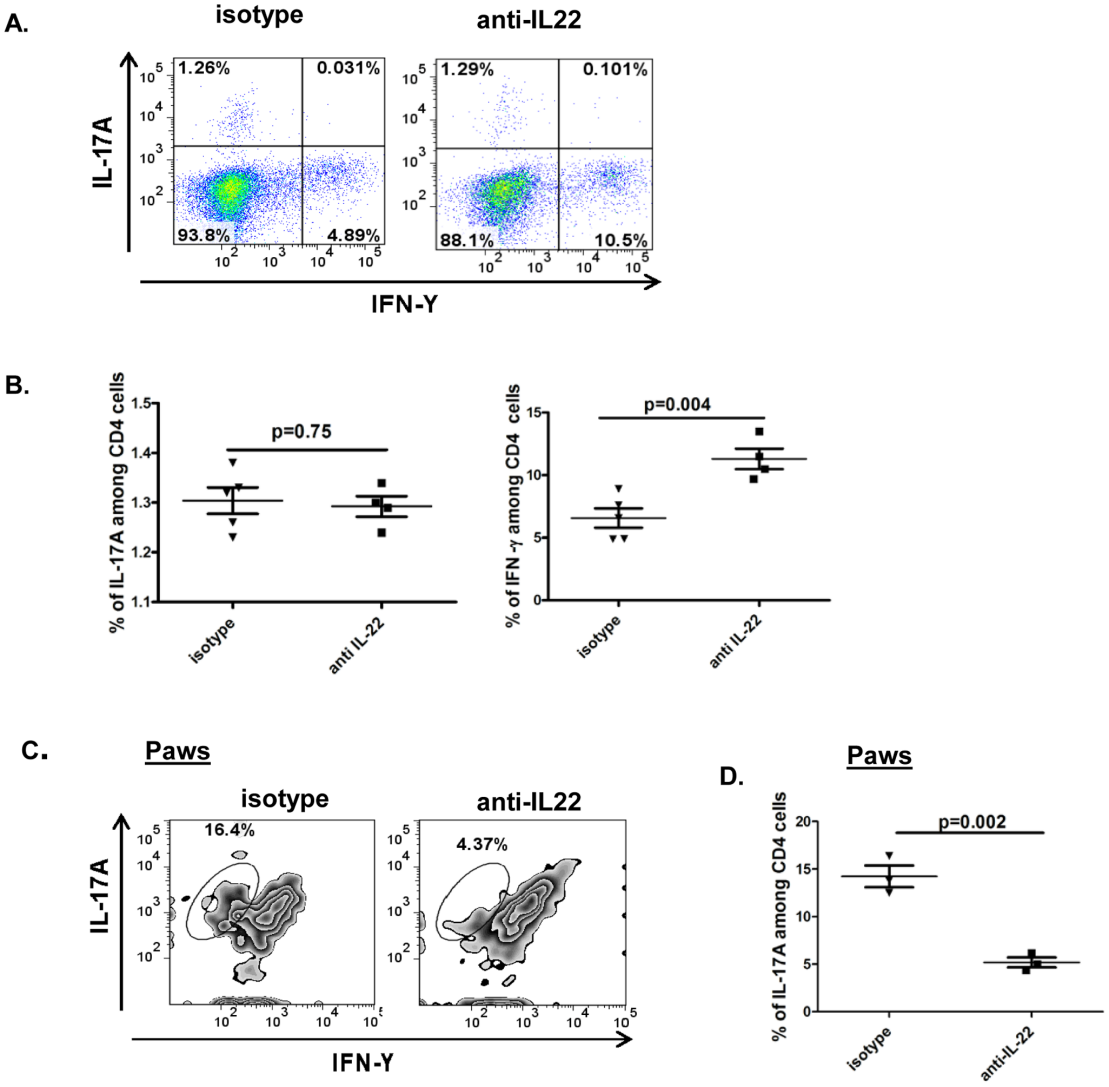
Neutralization of IL-22 after onset of arthritis preferentially increases the frequency of Th1 cells in lymphoid organs but not in joints. Experimental design is similar to figure 2. Draining inguinal lymph node cells and single cell suspensions of the paws from mice receiving anti-IL-22 antibody or isotype control were briefly stimulated ex-vivo with PMA/ionomycin and Brefeldin A for 6 hours followed by fluorescent labeling for surface anti-CD4 antibody and intra-cellular anti-IL-17A and anti-IFN-γ antibody. **A:** Representative dot plots shows IFN-γ and IL-17A staining on gated CD4 cells from lymph nodes. **B:** Percentages of CD4^+^IL-17^+^ or CD4^+^IFN-γ^+^ lymph node cells from the two groups of mice were plotted as a dot plot with each dot representing an individual mouse. **C:** Single cell suspension of joint cells from mice receiving anti-IL-22 antibody or isotype control were briefly stimulated ex-vivo with PMA/ionomycin and Brefeldin A for 6 hours followed by fluorescent labeling for surface anti-CD4 antibody and intra-cellular anti-IL-17A and anti-IFN-γ antibody. Representative zebra plots show IFN-γ and IL-17A staining on gated CD4 cells from paws. **D:** Percentages of CD4^+^IL-17^+^ cells from the paws of two groups of mice were plotted as a dot plot with each dot representing an individual mouse.

Several studies have established the role of Th1 and Th17 cells in CIA [21], [22], [23]. In order to elucidate the contribution of Th1 and Th17 cells to IFN-γ and IL-17 responses observed in figure 3, we undertook ex-vivo studies on lymphocytes from mice receiving anti-IL-22 or isotype control antibody. Data in figure 4A shows that Th1 response (CD4+IFN-γ+) was significantly increased in mice receiving anti-IL-22 (10.5%) vs mice receiving isotype control (4.89%). There was no change in the frequency of Th17 (CD4+IL-17A+) cells between the 2 groups. Figure 4B shows that the increase in Th1 cells is robustly significant at p = 0.004. Thus, the increased Th1 response with unaltered Th17 response seen by flowcytometry corroborated with the ELISA results in figure 3B and 3C.

Mice receiving anti-IL-22 antibody had lower arthritic scores than mice receiving isotype control antibody. In order to elucidate the joint specific Th1 and Th17 responses, single cell suspensions of arthritic joints were stained for CD4, followed by intracellular staining for IL-17 and IFN-γ. Data in figure 4C and 4D shows that mice receiving anti-IL-22 antibody had lower percentages of CD4+IL-17A+ cells in comparison to mice receiving isotype control. IFN-γ+ cells were not detectable in joints from either group of mice. Target organ inflammation is associated with the presence of increased numbers of Th17 cells in the affected organ. These findings are in keeping with the reduced severity of arthritis seen in mice receiving anti-IL-22 antibody.

To conclude, our data shows that reduced arthritis seen with anti-IL-22 antibody is associated with increased systemic Th1 responses and unaltered systemic Th17 responses.

### Increased expression of IL-22R1 on CD4 cells from arthritic mice and regulation of IFN-γ responses in T cells by IL-22

**Figure 5:**
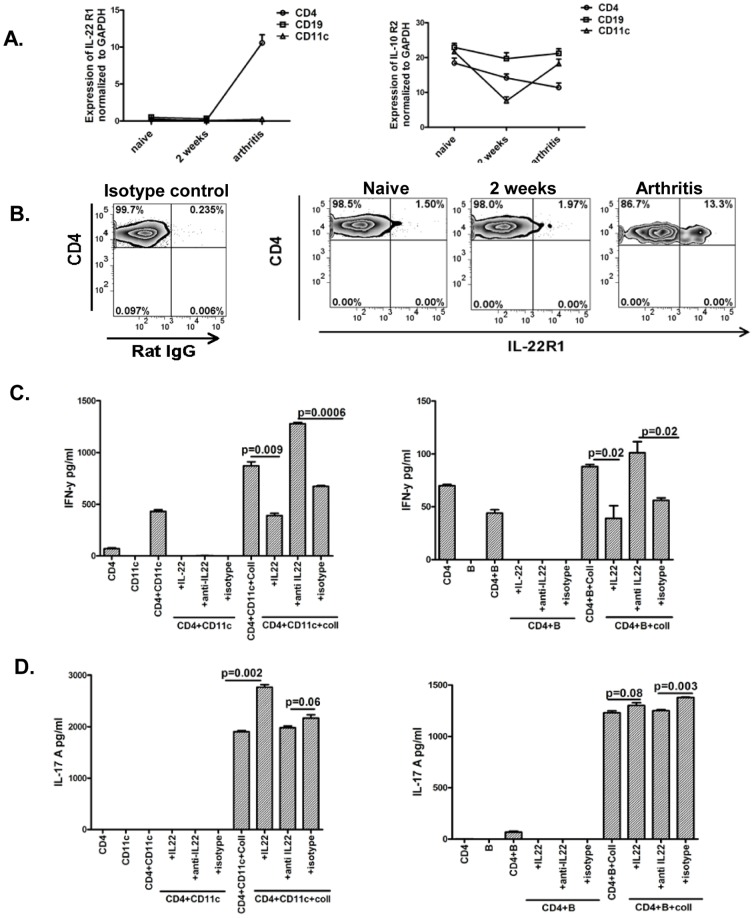
Increased expression of IL-22R1 on CD4 cells from arthritic mice and regulation of IFN-γ responses in T cells by IL-22. **A:** Expression of IL-22R1 and IL-10R2 were analyzed by realtime PCR in CD4+, CD19+, and CD11c+ cells enriched from splenocytes from various phases of arthritis. GAPDH was used as internal control. Data represented as fold change over GAPDH expression. Data is representative of 3 independent experiments. **B:** Splenocytes from various phases of arthritis were stained for CD4, and IL-22R1 and analyzed by flowcytometry. RatIgG was used as isotype control. Data shown is gated on mononuclear cells based on forward and side scatter followed by gating on CD4+ cells. Data is representative of 3 independent experiments. **C& D:** CD4 T, CD11c, and B cells were enriched from splenocytes of arthritic mice and co-cultured with recombinant IL-22 (100 ng/ml, Insight Genomics, USA), or anti-IL-22 antibody (10 ug/ml) or mouse isotype control (10 ug/ml, Biolegend) and/or collagen for 7 days. Supernatants were analyzed for IFN-γ (Fig. 5C) or IL-17A (Fig. 5D) by ELISA. Data is representative of two independent experiments.

IL-22 receptor is a heterodimeric complex of IL-22 receptor (IL-22R1) and a shared subunit, the IL-10 receptor (IL-10R2 or IL-10Rβ) of IL-10, IL-26 and IL-28/IL-19 [2].To elucidate the mechanism underlying augmented Th1 responses associated with administration of anti-IL-22 antibody we undertook studies to evaluate the expression of IL-22R1 and IL-10R2 during arthritis. Data in figure 5A shows that the level of expression of IL-22R1 on CD4+ cells is augmented in mice with arthritis, while naïve mice and mice from initiation phase have undetectable levels of this receptor. This is in contrast to CD19+ and CD11c+ cells which have undetectable levels of this receptor during the various phases of arthritis by gene expression analysis. Additionally, in keeping with constitutive and universal expression of IL-10R2 there is similar levels of expression of this subunit in CD4+, CD11c+ and CD19+ cells from various phases of arthritis (Fig. 5A). Increased expression of IL-22R1 on CD4+ cells from arthritic mice was confirmed with flow cytometry. Figure 5B shows the increased expression of IL-22R1 on CD4+ cells from arthritic mice (13.3%) in comparison to naïve mice (1.50%) or mice from the initiation phase (1.97%). These findings suggest that IL-22R1 may be upregulated on CD4+T cells during arthritis.

Our studies, so far, showed that neutralization of IL-22 in-vivo is associated with reduced severity of arthritis, increased frequency of Th1 cells and unaltered Th17 cells. Further there is induction of IL-22R1 on CD4+ cells during arthritis. We wanted to confirm the consequences of ligation of IL-22/IL-22R1 on CD4+ cells in the context of Th1 and Th17 responses. CD4+ cells from splenocytes of arthritic mice were co-cultured with CD11c+ cells or CD19+ cells in the presence or absence of recombinant IL-22, or anti-IL-22 antibody, or mouse IgG. Recombinant IL-22 significantly and robustly suppressed IFN-γ and anti-IL-22 augmented IFN-γ in CD4 T cell co-cultured with CD11c cells (Fig. 5C). Such an effect was also seen in CD4 T cell co-cultured with B cells, although with a reduced intensity probably because B cells are less potent antigen presenting cells in comparison to CD11c cells. Figure 5D shows that there is a significant effect on IL-17 responses under similar culture conditions, IL-22 inducing IL-17 in co-cultures of CD4 T cells and CD11c cells and anti-IL-22 significantly but modestly suppressing IL-17 in co-cultures of CD4 T and B cells. These findings provide evidence that T cells may respond to IL-22 during inflammation.

### The protective effect of anti-IL-22 is reversed in IFN-γ deficient mice

**Figure 6:**
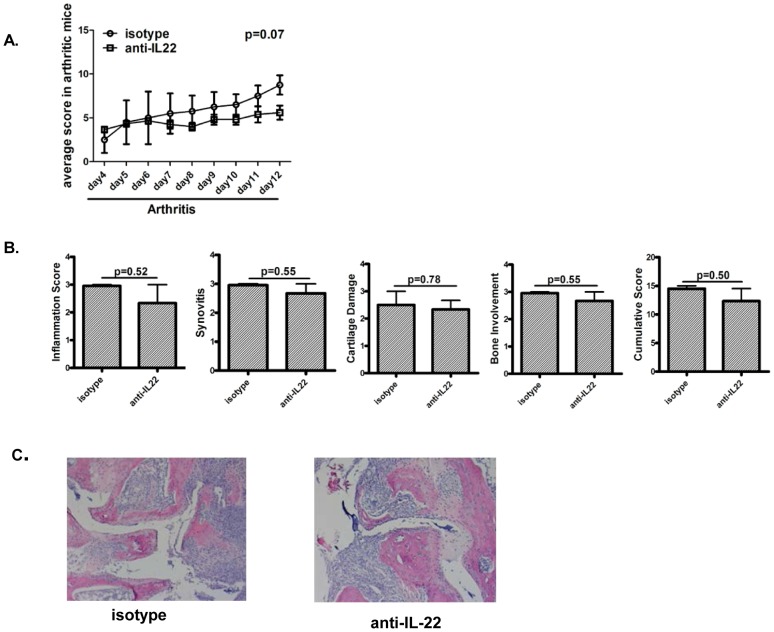
The protective effect of anti-IL-22 is reversed in IFN-γ deficient mice. **A:** 16 female B6.129S7-ifng^tm1Ts^ (B6.IFNγKO) mice were immunized with collagen and CFA. Mice with total score ≥1 and arthritis duration of ≥4 days were randomized to receive anti-IL-22 antibody (7–8 mice) or isotype control (7–8 mice) intraperitoneally at 100 ug/mouse/day for 8–9 days. Mice were scored for clinical arthritis every other day. Data shows cumulative arthritis score in 2 groups over time. **B:** Histologic scoring of paws for various aspects of joint damage and inflammation assessed after H&E staining. Data is from 4 mice per group. **C:** Hematoxylin and eosin staining of representative paws from the two groups of mice, magnification of 10×.

So far our data showed that administration of anti-IL-22 after onset of arthritis is associated with reduced severity of arthritis and increase in IFN-γ responses. In order to elucidate if the protective effect of anti-IL-22 is dependent on IFN-γ, we induced arthritis in IFN-γ deficient mice. Arthritic mice with scores of > or = 1 and arthritis duration of 4 days were randomized to receive anti-IL-22 antibody or isotype control for a total of 8–9 days. Data in figure 6A shows that the protective effect of anti-IL-22 is blunted in IFN-γ deficient mice. In keeping with the clinical scoring, histology of the joints showed similar scores of inflammation, synovitis, cartilage destruction and bone involvement in mice receiving anti-IL-22 antibody or isotype control antibody (Fig. 6B and C).

### Neutralization of IL-22 prior to onset of arthritis increases incidence and severity of arthritis

**Figure 7:**
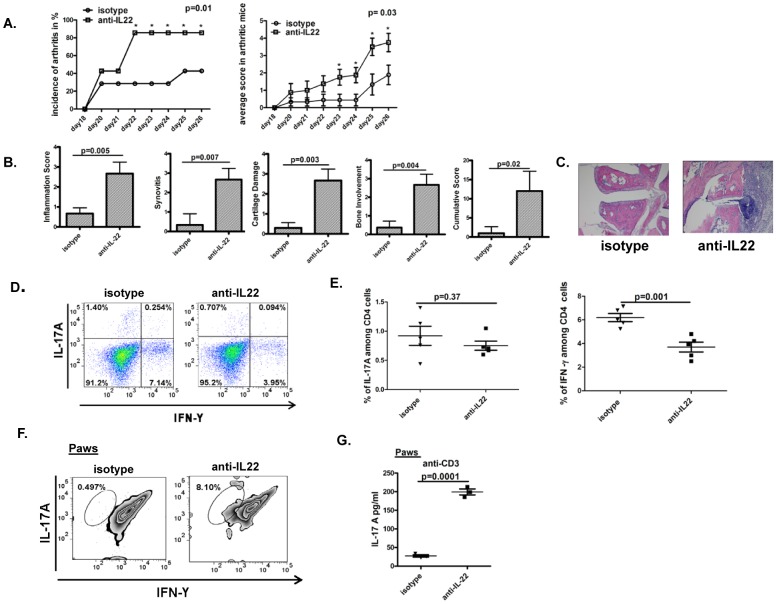
Neutralization of IL-22 prior to onset of arthritis increases incidence and severity of arthritis. **A:** 16 DBA mice were immunized with collagen and CFA. On day 18 following immunization, mice were divided into 2 groups (8 mice per group) to receive either anti-IL-22 antibody (100 ug/mice/day) or isotype control (100 ug/mouse/day) intraperitoneally for 8–10 days. Mice were scored for clinical arthritis every other day. Data shows incidence and cumulative arthritis score in 2 groups over time. **B:** Histologic scoring of paws for various aspects of joint damage and inflammation assessed after H&E staining. Data is from 4 mice per group. **C:** Hematoxylin and eosin staining of representative paws from the two groups of mice, magnification of 10×. **D:** Draining inguinal lymph nodes cells from mice receiving anti-IL-22 antibody or isotype control were briefly stimulated ex-vivo with PMA/ionomycin and Brefeldin A for 6 hours followed by fluorescent labeling for surface anti-CD4 antibody and intra-cellular anti-IL-17A and anti-IFN-γ antibody. Representative dot plots shows IFN-γ and IL-17A staining on CD4 gated cells. **E:** Percentages of CD4^+^IL-17^+^ cells or CD4^+^IFN-γ^+^ cells from lymph nodes from the two groups of mice were plotted as a dot plot with each dot representing an individual mouse. **F:** Single cell suspension of joint cells from mice receiving anti-IL-22 antibody or isotype control were briefly stimulated ex-vivo with PMA/ionomycin and Brefeldin A for 6 hours followed by fluorescent labeling for surface anti-CD4 antibody and intra-cellular anti-IL-17A and anti-IFN-γ antibody. Representative zebra plots show IFN-γ and IL-17A staining on gated CD4 cells from paws. **G:** Single cell suspensions of the joint cells from mice receiving anti-IL22 or isotype control were stimulated with anti-CD3 (5 ug/ml for 3 days) and IL-17A was measured in culture supernatants by ELISA. Each dot represents an individual mouse.

Previously, we have reported that administration of recombinant IL-22 prior to onset of arthritis is associated with reduction in the severity of arthritis, suggestive of a protective role prior to onset of arthritis. In this study we report that neutralization of IL-22 after onset of arthritis reduces severity of arthritis, implying a pathogenic role after onset of arthritis. In order to elucidate the role of neutralizing IL-22 before onset of arthritis, anti-IL-22 antibody or isotype control was administered to mice from day 18 following immunization with collagen for a total of 8–10 days. Mice receiving anti-IL-22 had increased incidence and severity of arthritis (Fig. 7A). Histopathologic examination of the paws showed increased scores of inflammation, synovitis, cartilage destruction and bone involvement in mice receiving anti-IL-22 antibody (Fig. 7B and C). Ex-vivo analyses of CD4+IL-17+ or CD4+IFN-γ+ cells from lymphocytes of mice from either group showed that the frequencies of Th17 remained the same between the two groups (Fig. 7D&E). Th1 frequency was lower in mice receiving anti-IL-22 antibody in comparison to mice receiving isotype control (Fig. 7D&E). In order to elucidate the joint specific Th1 and Th17 responses, single cell suspensions of arthritic joints were stained for CD4, followed by intracellular staining for IL-17 and IFN-γ. Data in figure 7F shows that mice receiving anti-IL-22 antibody had higher percentages of CD4+IL-17A+ cells in comparison to mice receiving isotype control. IFN-γ+ cells were not detectable in joints from either group of mice. Single cell suspensions of joints from mice receiving anti-IL-22 or isotype control were also stimulated with anti-CD3 for 3 days or collagen for 7 days. Significantly higher levels of IL-17A was detectable in joint cells of mice receiving anti-IL-22 in comparison to isotype control when stimulated with anti-CD3 (Fig. 7G) and IL-17A was undetectable when joints cells were stimulated with collagen (data not shown). IFN-γ remained undetectable whether joint cells were stimulated with anti-CD3 or collagen (data not shown). These findings support a protective role of IL-22 prior to onset of arthritis.

## Discussion

CD4 T, NK and LTi-like cells can produce IL-22 [1]. In CIA, IL-22 response increases with onset of joint inflammation and is produced primarily by CD4 T cells or CD49b cells [20]. Our data shows that collagen specific IL-22 response expands with onset of arthritis and IL-22 producing cells are discrete from IL-17 or IFN-γ producing cells. Although some studies suggest the presence of cells co-producing IL-17 and IL-22, our findings are in keeping with studies in inflammatory diseases such as psoriasis, uveitis, atopic dermatitis, ankylosing spondylitis and RA reporting the presence of IL-22 producing cells which are distinct from IL-17 producing cells [24], [25], [26], [27]. In this report we show that administration of neutralizing antibody to IL-22 after onset of arthritis is associated with reduction in severity of arthritis, consistent with a pathogenic function of IL-22 during arthritis. This pathogenic effect of IL-22 is in keeping with reduced incidence of arthritis in IL-22 deficient mice [19]. Current evidence suggests that IL-22 plays a pathogenic role in RA, in that IL-22 levels are increased in serum and synovial fluid of patients with RA compared to controls, IL-22 induced RANKL and the increased levels of IL-22 correlated with disease activity and bone erosions [14], [15], [16], [17], [18].

IL-22 deficient mice had similar degree of arthritis as wild type mice in a model where joint inflammation was induced after local injection of mBSA into joints, suggesting that IL-22 is not pathogenic in the absence of prolonged systemic inflammation [28]. In the IL-1receptor antagonist deficient mice, neutralization of IL-22 after arthritis onset reduced bone damage without any change in overall inflammation [29]. Furthermore, administration of recombinant IL-22, prior to the onset of arthritis, reduces the progression to severe arthritis in the model of CIA [20]. In a preclinical model of uveitis IL-22 is protective when administered prior to the onset of inflammation. Such an effect is not seen when IL-22 was administered after disease onset [30]. In our current study we show that neutralization of IL-22 prior to onset of joint inflammation, augments arthritis supporting a protective role of IL-22 during this phase. These findings point to the dual nature of the effector function of IL-22 depending on several factors, including the makeup of the inflammatory mileu, as well as degree and duration of inflammation. In fact, in a model of airway inflammation IL-22 was shown to be protective in the absence of IL-17 and pathogenic in the presence of IL-17 [4].

The balance of systemic Th17/Th1 response plays a decisive role in CIA. In CIA where only 60–70% of mice develop arthritis following a single immunization with collagen and CFA, an increased systemic Th17/Th1 response is observed only in mice developing arthritis. Mice that do not develop arthritis following immunization do not have increased systemic Th17/Th1 responses [21]. Furthermore, IFN-γ plays a protective role in arthritis and deficiency of IFN-γ results in increased arthritis which is abrogated by neutralizing IL-17 [31], [32], [33], [34], [35], [36], [37], [38]. In our study, mice receiving anti-IL-22 antibody had increased IFN-γ response to collagen re-stimulation with increased frequency of Th1 cells. IL-17 production in response to collagen re-stimulation and frequency of Th17 cells ex-vivo remained unchanged. This is in keeping with the overall protective role of IFN-γ in CIA and a recent study reporting selective deficiency of Ahr, a transcription factor for IL-22, in CD4 T cells being associated with reduced arthritis and increased Th1 response but an unaltered Th17 response [39].

In our study, although we could not detect IFN-γ in arthritic joints, systemic levels of IFN-γ probably play an important role in modulating inflammation in the target organs. Several studies support the protective effect of IFN-γ via regulation of other systemic immune molecules. IFN-γ confers protection by regulating systemic neutrophil–attracting chemokine, granulocyte chemotactic protein-2 (GCP-2), thus preventing neutrophil influx into joints [40]. Furthermore, IFN-γ has been shown to mediate its protective effect by regulating accumulation of CXCR2 expressing neutrophil in joints [33]. Additionally, IFN-γ mediates its protective effect in CIA by the regulation of proinflammatory cytokine IL-1β [34]. The protective role of increased systemic IFN-γ in the presence of anti-IL-22 is confirmed in our study, where administration of anti-IL-22 antibodies to IFN-γ deficient arthritic mice resulted in blunting of the protective effect of anti-IL-22.

IgG2a and IgG2b anti-collagen antibodies are pathogenic in the induction of joint inflammation in CIA. In keeping with the therapeutic effect of anti-IL-22, when administered after the onset of arthritis, IgG2a anti-collagen antibodies are significantly but modestly reduced and IgG1 and IgG2b antibodies are unaltered in mice receiving anti-IL-22. The protective effect of anti-IL-22 is blunted in IFN-γ deficient mice, indicating that increased IFN-γ responses associated with the administration of anti-IL-22 plays a dominant role behind its protective function during arthritis. The contribution of reduced IgG2a responses in mediating the protective function of anti-IL-22 could not be evaluated in IFN-γ deficient mice since these mice have very low levels of IgG2a. The contribution of IgG2a responses in IL-22 mediated regulation of inflammation as well as the direct or indirect mechanisms of IL-22 mediated regulation of B cell responses remains to be elucidated.

Previous studies have shown that IFN-γ induces IgG2a production in B cells. This effect of IFN-γ on B cells is dependent on co-stimulation via another pathway including TLR, surface immunoglobulin+IL-1+IL-2 and independent of CD40 pathway [41], [42], [43]. Interestingly CD40L deficient mice have reduced levels of IgG2a [44]. These findings point to the intricate regulation of the IgG2a response in vitro and in vivo. In our studies we find that administration of anti-IL-22 is associated with expansion of IFN-γ responses in conjunction with modestly reduced (although significant) IgG2a responses. It is possible that anti-IL-22 may have effects on the expression of other known (TLR, surface immunoglobulin, IL-1, IL-2) as well as unkown molecules necessary for the regulation of IgG2a responses in B cells, which may explain the low levels of IgG2a along with high IFN-γ levels in-vivo.

IL-10 plays a protective role in inflammation and the protective function of IL-22 prior to onset of inflammation is associated with an augmented IL-10 response [20], [30]. IL-10 response in mice receiving anti-IL-22 after the onset of arthritis is not significantly different from mice receiving isotype control suggesting that IL-22 mediated modulation of IL-10 is dependent on the overall level of inflammation: being blunted in the presence of high levels of inflammation. IL-22 receptor is a heterodimeric complex of IL-22 receptor (IL-22R1) and the shared IL-10 receptor (IL-10R2 or IL-10Rβ) of IL-10, IL-26 and IL-28/IL-19 [2]. IL-10R2 is ubiquitously expressed, whereas, IL-22R1 is highly expressed on non-immune cells in skin, colon, small intestine, pancreas, kidney, and liver [45]. Past studies done in naïve Balb/c mice (this specific strain of mice is resistant to autoimmune inflammation) in the absence of stimulation or after short in-vivo stimulation with LPS, or on lymphocytes from healthy volunteers report that IL-22R1 is not expressed on lymphocytes during homeostasis or following brief stimulation [45], [46], [47]. Studies with differentiated Th1 and Th2 cells have reported the IL-22 mediated regulation of IFN-γ and IL-4 [48]. In another study, prolonged stimulation of human T cells in the presence of IL-22 decreased IFN-γ, increased IL-4 and IL-13, and had no effect on IL-10 [49]. Studies have reported that T cells from anaplastic lymphoma and some B cell lymphoma lines may express this receptor, and forced expression of IL-22R1 on T and B cells results in generalized inflammation in mice suggesting that the signaling machinery utilizing this receptor subunit is present on T and B cells [50], [51], [52], [53]. IL-22R1 is also expressed on CD11b+ cells in an animal model of autoimmune uveitis in inflammation susceptible C57BL/6 mice [30]. In keeping with the non-expression of IL-22R1 on immune cells, our studies showed, splenic CD4+ cells express negligible levels of IL-22R1 during homeostatic state and during the initiation phase following immunization with collagen. However, there is increased expression of this receptor subunit in splenic CD4+ cells after onset of arthritis. IL-10R2 which is constitutively expressed in immunocytes was expressed at comparable levels in naïve mice, mice from the initiation phase and arthritic mice. Flowcytometry analysis of the IL-22R1 on CD4+ cells also showed that the expression of this subunit is increased with the onset of arthritis, confirming the findings of real-time PCR studies. In order to elucidate the functional consequence of expression of the IL-22R1 on CD4+ cells, CD4+ cells from arthritic mice were cultured with antigen presenting cell and collagen in the presence and absence of recombinant IL-22 and neutralizing antibody to IL-22. In keeping with the in-vivo effects, in-vitro anti-IL-22 increased IFN-γ and recombinant IL-22 suppressed IFN-γ. These findings suggest that IL-22R1 expression on T cells during inflammation is associated with functional consequences, specifically alteration of IFN-γ regulation in T cells.

Thus, it is possible that IL-22R1 may be induced on immunocytes during inflammatory states and/or expression of IL-22R1 may be restricted to specific strains of mice which are susceptible to inflammation. Further studies are needed to elucidate the factors inducing this receptor subunit on immunocytes during inflammation and cancer. In summary our data shows, for the first time, that neutralization of endogenous IL-22 after onset of arthritis is associated with reduction in the severity of arthritis and administration of anti-IL-22 prior to onset of arthritis results in increased incidence and severity of arthritis. Furthermore, the protective function of anti-IL-22 is associated with unaltered Th17 responses and increased Th1 responses which are blunted in IFN-γ deficient mice. Additionally, CD4 cells may express IL-22R1 during inflammation thus enabling them to respond to IL-22.

## Supporting Information

Figure S1 **Joint specific IL-22, IL-17 and IFN-γ responses.** **S1A:** Single cell suspensions of the paws from arthritic mice was stimulated with anti-CD3 (5 ug/ml for 3 days) and IL-17A, IFN-γ or IL-22 were measured in culture supernatants by ELISA. Data is representative of 3 independent experiments with 3 mice per experiment. **S1B:** Single cell suspensions of the paws from arthritic mice were stimulated with PMA/ionomycin and Brefeldin A for 6 hours, stained intra-cellularly for IL-17, IFN-γ or IL-22 and analyzed by flow-cytometry. Data shown is gated on mononuclear cells based on forward and side scatter. Data is representative of 3 independent experiments with 3 mice per experiment. **S1C:** Single cell suspensions of the paws from arthritic mice was stimulated with PMA/ionomycin and Brefeldin A for 6 hours, followed by fluorescent labeling for surface anti-CD4 antibody and intra-cellular anti-IL-17A and anti-IL-22 antibody. Data shown is gated on CD4 cells. Percentages of CD4^+^IL-17^+^ cells or isotype control for IL-17 were plotted as dot plot with each dot representing an individual mouse. Data is representative of 3 independent experiments with 3 mice per experiment.
(TIF)
