# Supplementary figures and images for: Endogenous IL-22 Plays a Dual Role in Arthritis: Regulation of Established Arthritis via IFN-γ Responses
Source: PLoS One. 2014 Mar 27;9(3):e93279. doi: 10.1371/journal.pone.0093279 (PMC3968131; doi:10.1371/journal.pone.0093279)

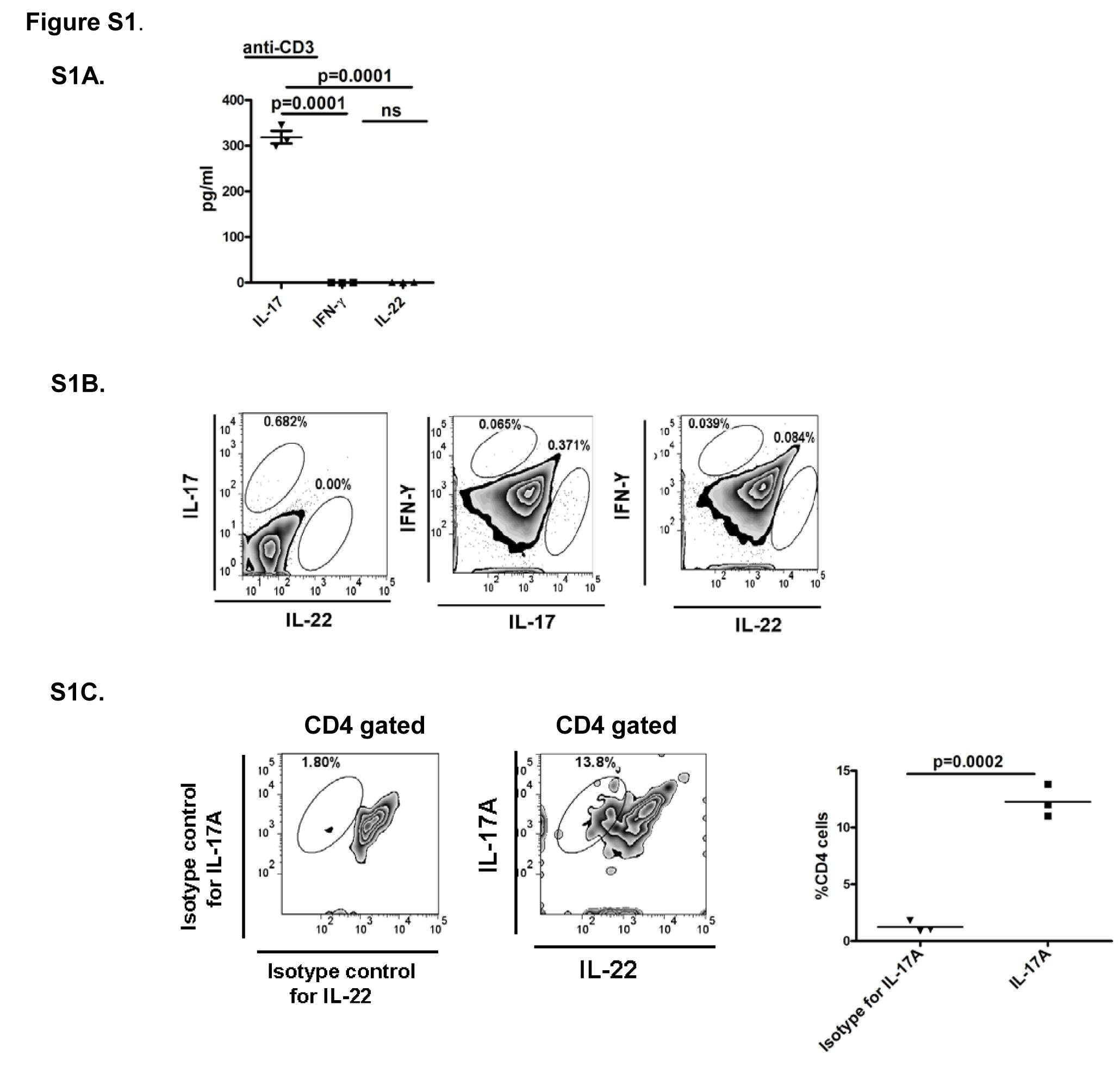

Supplement: Figure S1 — Joint specific IL-22, IL-17 and IFN-γ responses. S1A: Single cell suspensions of the paws from arthritic mice was stimulated with anti-CD3 (5 ug/ml for 3 days) and IL-17A, IFN-γ or IL-22 were measured in culture supernatants by ELISA. Data is representative of 3 independent experiments with 3 mice per experiment. S1B: Single cell suspensions of the paws from arthritic mice were stimulated with PMA/ionomycin and Brefeldin A for 6 hours, stained intra-cellularly for IL-17, IFN-γ or IL-22 and analyzed by flow-cytometry. Data shown is gated on mononuclear cells based on forward and side scatter. Data is representative of 3 independent experiments with 3 mice per experiment. S1C: Single cell suspensions of the paws from arthritic mice was stimulated with PMA/ionomycin and Brefeldin A for 6 hours, followed by fluorescent labeling for surface anti-CD4 antibody and intra-cellular anti-IL-17A and anti-IL-22 antibody. Data shown is gated on CD4 cells. Percentages of CD4+IL-17+ cells or isotype control for IL-17 were plotted as dot plot with each dot representing an individual mouse. Data is representative of 3 independent experiments with 3 mice per experiment. (TIF) [file pone.0093279.s001.tif]
